# First identification of *Gordonia sputi* in a post-traumatic endophthalmitis patient – a case report and literatures review

**DOI:** 10.1186/s12886-017-0573-5

**Published:** 2017-10-11

**Authors:** Wei Fang, Jiuke Li, Hu-Shan Cui, Xiaohong Jin, Jing Zhai, Yuanmin Dai, Yumin Li

**Affiliations:** Ophthalmology Department of SIR RUN RUN SHAW hospital, SIR RUN RUN SHAW Institute of Clinical Medicine of Zhejiang University, #3 Qingchun East Road, Hangzhou Zhejiang, 310016 People’s Republic of China

**Keywords:** Endophthalmitis, *Gordonia sputi*, *Actinomyces*, Traumatic, Case report

## Abstract

**Background:**

We present a case of post-traumatic endophthalmitis with relatively good prognosis caused by *Gordonia sputi,* which, to our knowledge is the first case in the literature.

**Case presentation:**

A 24 year old man, who underwent an intraocular foreign body extraction half a month before presentation in the left eye, was referred to us complaining of blurred vision and slight pain for 5 days. His first presentation showed moderate intracameral and intravitreous purulent inflammation with a best corrected vision of counting fingers. After gram staining of the intravitreous samples revealed a gram-positive bacilli infection, a combination of amikacin and vancomycin was initially injected intravitreously. The left eye kept stable for three days but deteriorated on the 4th day. On the 5th day after presentation conventional culture characterized the bacterium as an *Actinomyces sp.* while 16S ribosomal RNA gene sequencing confirmed it as *Gordonia sputi*. Thereby a complete pars plana vitrectomy combined with lensectomy and silicone oil tamponade was performed. During the surgery an intraocular irrigation with penicillin G was adopted, followed by administration of intravenous penicillin G twice one day for a week. A relatively normal fundus with slight intracameral inflammation was observed a week after the operation, and the best corrected vision recovered to 0.15. One year later his vision remained 0.1.

**Conclusion:**

*Gordonia sputi* should be taken into consideration in patients with post-traumatic endophthalmitis especially due to foreign body penetration. Compared to conventional laboratories, molecular methods are recommended for an accurate diagnosis. A comprehensive strategy of antimicrobial agents and vitrectomy may render a satisfactory result.

**Electronic supplementary material:**

The online version of this article (10.1186/s12886-017-0573-5) contains supplementary material, which is available to authorized users.

## Backgound

Post-traumatic endophthalmitis is a severe vision-threatening infection of intraocular tissues, accounting for 20–30% of the post-operative cases, while eyes with intraocular foreign bodies have a risk of infection about twice as high as those without a foreign body [1, 2]. Previous studies revealed about two-thirds to three quarters of the causative pathogen are gram-positive organisms [3]. Recently, one quarter of the infections were due to *bacillus spp.*, making this the second most common pathogen, especially in the eyes with intraocular foreign body, and most of them have a particularly poor prognosis [4, 5]. According to the previous literatures, only one-ten of the eyes reported had a final vision better than being able to count fingers [6]. Here we depict the clinical and pathogenic features of a rare intraocular gram-positive bacilli infection with relatively good outcomes, which, to our knowledge, is the first identification of *Gordonia sputi* in endophthalmitis. We declared that this study is in accordance with the tenets of Helsinki, approved by SIR RUN RUN SHAW hospital ethic committee and this patient has signed an informed consent.

## Case presentation

**Fig. 1:**
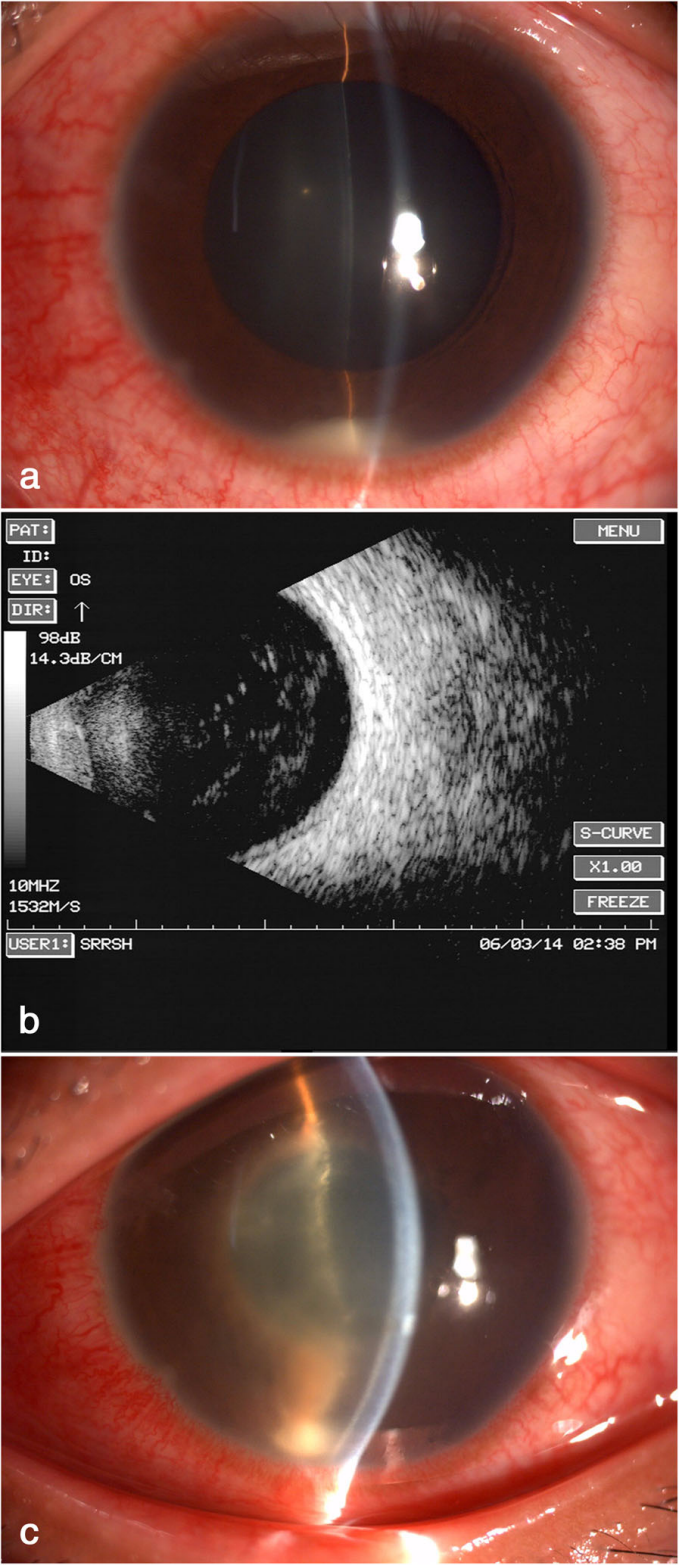
Pre-operative evaluation of the infected eye. **a** slitlamp examination at first presentation revealed moderate congestion in the left eye, with a transparent cornea, anterior cells4+, about two millimeters high hypopyon, a transparent lens. **b** ultrasonic B scan showed severe vitreous opacity without any posterior vitreous detachment. **c** after keeping stable for three days with vitreous injection of antibiotics, his left eye deteriorated obviously on the 4th day, with a vision of hand movement and an unobservable fundus

**Fig. 2:**
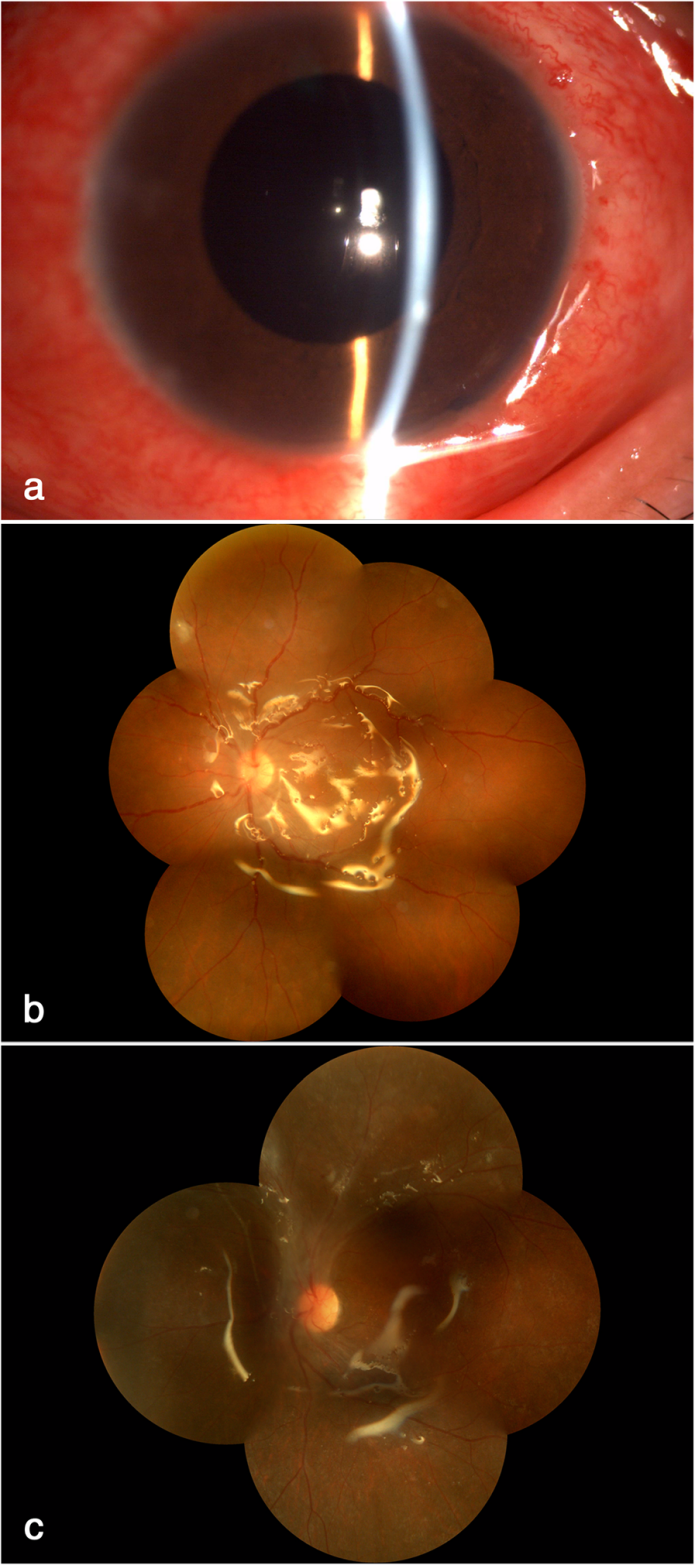
Post-operative evaluation of the infected eye. **a-b** one week after operation, a relatively normal fundus with slight intracameral inflammation was observed, with a best corrected vision of 0.15. **c** one year later the retina keep attached with slight fibrous proliferation beside optic head and the vision remained 0.1

A 24 year old man was referred to us complaining of blurred vision and slight pain in the left eye for 5 days. He underwent an intraocular foreign body (an iron splinter from a working lathe) extraction at a local hospital half a month before presentation. The foreign body was extracted from the corresponding pars plana incision using an electromagnetic tip. His right eye’s best corrected vision was 0.9 while his left eye’s vision was counting fingers at 20 cm, with a normal intraocular pressure. Slitlamp examination revealed moderate congestion in the left eye, with a transparent cornea, anterior cells4+, about two millimeters high hypopyon, a transparent lens and multiple clusters of white coalescent lesions on the fundus, while the large vessels of retina could still be obscurely observed (Fig. 1a). B scan showed a severe vitreous opacity without any posterior vitreous detachment (Fig. 1b). So, a diagnosis of infectious endophthalmitis was presumed and vitreous samples were immediately obtained for pathogenic identification. Contemporary gram stain showed a gram-positive bacilli infection, so empirically 0.1 ml of amikacin (400μg) combined with vancomycin (0.8 mg) was injected intravitreally and topical 0.5% levofloxacin eye drops 4 times one day were administered. After keeping stable for three days, his left eye deteriorated obviously on the 4th day, with a vision of only hand movement and an unobservable fundus (Fig. 1c). On the 5th day, the conventional cultures characterized the bacterium as an *Actinomyces sp.* on the basis of colony morphology and conventional biochemical reactions. Meanwhile 16S ribosomal RNA (rRNA) gene sequencing was conducted using a pair of universal primers. The obtained product sequences (1408 bp) were compared with published sequences in the GenBank database (http://www.ncbi.nlm.nih.gov/blast), which confirmed the pathogen as *Gordonia sputi* (*Gordonia sputi* strain IFM 10747 16S rRNA gene, partial sequence, Sequence ID: gb|FJ536318.1|, 100% similarity). Therefore, a complete pars plana vitrectomy combined with lensectomy and silicone oil tamponade was performed. Massive white purulent clusters in the vitreous body and on the retinal surface with huge retinal breaks in the peripheral retina were found during the surgery. An intraocular irrigation with penicillin G (a concentration of 80μg/ml) was adopted. Post-operative administration included intravenous penicillin G 4 million units twice one day for a week, topical 0.5% levofloxacin and 1% prednisolone acetate eye drops 4 times one day for two weeks. A relatively normal fundus with slight intracameral inflammation was observed a week after the operation (Fig. 2a-b), and the best corrected vision recovered to 0.15. One year later the retina remained attached with slight fibrous proliferation beside the optic head and his vision remained 0.1 (Fig. 2c).

## Discussions

Members of genus Gordonia are widely distributed in nature, and about 29 species have been identified. From 1996 to 2015, only 16 cases of infections caused by *Gordonia sputi* were reported worldwide, most of which were catheter related, such as contaminated central venous catheters and chest tubes, in a setting of immunocompromised status [7–9]. *Gordonia spp.* infection usually has a subacute or chronic course, sometimes resembling fungi infection. The patient in this case presented with vision blurred about 10 days after the iron foreign body penetrating, showing multiple clusters of white purulent lesions in the anterior chamber, vitreous cavity and on the retina, without obvious pain, which might clinically indicate a less virulent bacteria or fungi infection. According to the contemporary gram stain and its reaction to intravitreal antibiotic a gram-positive bacilli infection was presumed, and further molecular examinations confirmed the pathogen as *Gordonia sputi.* So, when facing a subacute course of intraocular infection after foreign body penetration the pathogen of *Gordonia spp.* should be taken into consideration.

Conventional tests of the cultured pathogenic samples usually misidentify *Gordonia spp.* as other gram-positive bacilli, such as *Rhodococcus* or *Nocardia spp.* However, so far bacterial rRNA gene sequencing analysis has proven to be a more accurate measurement [10]. As Lai CC et al. reported, in a group of 66 isolates previously identified as *Rhodococcus spp.* by conventional microbiological and biochemical analysis, 15 isolates were found to be Gordonia spp. by 16S rRNA gene sequencing [7]. These findings suggest that *Gordonia spp.* may be severely underreported. Meanwhile because genomic sequencing is a common and inexpensive laboratory technique, we suggest that, for early diagnosis, genomic sequencing for *Gordonia* species should be taken actively when patients present with gram-positive bacilli related infections.

The regimen of endophthalmitis therapy includes antimicrobial agents and vitrectomy. According to the valid literatures, *Gordonia sputi* is susceptible to most antibiotics during E test for *Nocardia* species and other *actinomycetes*, like carbapenems, cephalosporins, aminoglycosides, new fluoroquinolones and vancomycin [8, 9]. However, the resistance of *Gordonia spp.* appears to increase recently. For instance, *Gordonia spp.* exhibits about 11% resistance to vancomycin in a report by Blaschke AJ [11]. In this case, an intravitreal injection of amikacin combined with vancomycin was empirically chosen first and no deterioration was observed in the early three days. Nevertheless, the patient got worse on the 4th day maybe due to the pharmacokinetics of intravitreous injection so a vitrectomy was arranged then. As we all know, vitrectomy plays an important role in the therapy against endophthalmitis, which could almost entirely clear the necrotic intraocular tissues, causative microorganism, and help to irrigate the vitreous cavity with effective anti-microorganism agents. In addition to use as initial therapy, vitrectomy should be considered for eyes poorly responding to an original anti-microorganism strategy. In this case, we performed the complete vitrectomy and lensectomy after definite identification of the pathogen so the corresponding antibiotics could be chosen during operation accordingly. It seems to render a satisfactory visual and anatomical outcome from this combined treatment strategy.

## Conclusions

*Gordonia sputi* should be taken into consideration in patients with post-traumatic endophthalmitis due to foreign body penetration. Compared to conventional laboratories, molecular methods are recommended for an accurate diagnosis. A comprehensive strategy of antimicrobial agents and vitrectomy may render a satisfactory result.

## Additional file

Additional file 1: A 16s rRNA sequence of the obtained pathogenic bacteria. (TXT 1 kb)

## Abbreviations

rRNA: Ribosomal ribonucleic acid
